# Measuring 3D shape in orthodontics through geometric morphometrics

**DOI:** 10.1186/s40510-017-0194-9

**Published:** 2017-12-01

**Authors:** Luis Huanca Ghislanzoni, Roberta Lione, Paola Cozza, Lorenzo Franchi

**Affiliations:** 1 Department of Orthodontics, University of Geneve, Geneve, Switzerland; 2 Department of Orthodontics, University of Rome Tor Vergata, Rome, Italy; 3 Department of Surgery and Translational Medicine, University of Florence, Florence, Italy; 4 Avenue Belles-Roches 7, 1004 Lausanne, VD Switzerland

## Abstract

**Background:**

Geometric morphometrics (GMM) has been traditionally applied to the field of biology to study developmental differentiations between species. Orthodontics deals with the shape and size of the face and its components. While several tools have been used to measure size, proportions, and relations between anatomical components, shape has been mainly described by esthetic criteria. The purpose of this paper is to present methods to measure shape of 3D orthodontic data, beyond the conventional tools that have been traditionally used in cephalometrics and in facial and dental cast analysis.

**Findings:**

The authors showcase an example of applying geometric morphometrics to measure palates from scanned dental casts. GMM can be used as a useful tool to describe the three-dimensional shape of surfaces of orthodontic interest. A general introduction to the theoretical principles of how to apply GMM is provided.

**Conclusions:**

Variability can be measured through the Principal Component Analysis (PCA) and can lead to the identification of shape patterns and sources of variability of the shape, independently from changes in size.

## Introduction

Technological advances have made three-dimensional (3D) orthodontic diagnostic records much more accessible. We now have 3D images of the craniofacial skeleton from CBCT imaging, 3D facial photographs from stereophotogrammetry camera sets, and dental casts from intraoral and standard scanner, all available in digital format. We are, therefore, in the condition to measure and evaluate what interests us most as orthodontists, namely shape, in ways that were not previously possible [1, 2]. Potentially, this is a turning point in orthodontic diagnosis. Since 3D data are not a mere extension of 2D data to an extra dimension but require new tools to fully exploit their 3D nature, we can now grasp this opportunity and radically overhaul our diagnostic armamentarium.

Geometric morphometrics (GMM) [3–6] has been traditionally applied to the field of biology to study developmental differentiations between species [7, 8], and several researchers applied this method to orthodontics [9–14]. Orthodontics deals with the shape and size of the face and its components. While several tools have been used to measure size, proportions, and relations between anatomical components, shape has been mainly described by esthetic criteria. GMM can be used as a specific tool to describe shape variation between individuals and to identify patterns of variation of orthodontic interest (es. hypo/hyper-divergency or sagittal relationship of the basal bones) [14–17]. When applied to 2D cephalometrics, it can provide apparently surprising results, eg., the main source of difference between patients classified as Class I and Class II consists of the vertical growth pattern, rather than the expected difference along the sagittal plane [12]. GMM can be useful to understand shape variation, especially in 3D where the shape complexity gets to its maximum [9, 15].

The purpose of this paper is to present methods to measure the shape of 3D orthodontic data, beyond the conventional tools that have been traditionally used in cephalometrics and in facial and dental cast analysis. The authors showcase an example of applying geometric morphometrics to measure palates [9] from scanned dental casts.

## Geometric morphometrics principles

GMM is a special method to measure shape, as it does not use traditional angles and linear distances (size measures). By choosing specific angles or linear measurements, we arbitrarily choose which part of the shape to measure. In fact, the selection of some measurements and exclusion of others can lead to biased result as you consider just one specific part of the shape, instead of the whole of it. With GMM, the whole of the shape can be analyzed.

The basic principles of GMM are the following: [5, 18, 19]

- An object is a collection of landmarks. Objects such as teeth, bones, and faces need to be reduced to a set of landmarks before analysis can proceed, as the basic tools of GMM tools cannot work directly on curves or surfaces. Objects of the same class (e.g., faces) must have the same number of landmarks and the landmarks must be homologous, i.e., each landmark must represent the same anatomical (or functional) feature.
- Shape of an object can be measured only in relation to another object, i.e., shape measurement is actually the comparison between two shape/measurement of shape difference.
- The “shape” is the morphologic entity that remains after position, and size differences have been removed from the analyzed objects (as dimensional differences are not considered when comparing shapes).

Practically, when analyzing a collection of objects, the main aim is to find the *average* shape of the objects and then analyze the *variability* of shape in the group, with regard to the average shape. The variability (i.e., how much shape varies between the objects and in which ways) is analyzed through the Principal Component Analysis (PCA) statistical procedure. In a biological system such as the craniofacial complex, shape variability can be clinically translated into shape patterns (e.g., the dolichofacial–brachyfacial pattern, or the Class II–Class III anteroposterior pattern). The patterns that are most dominant are usually identified by clinical experience. GMM can reveal these patterns and measure the relative contribution of each to the total shape variability.

## From Cartesian coordinates to the shape–space system

The procedure that GMM follows to compute average shape and shape variability is a sequence of the following actions: [18]

- Landmarks are placed on the objects at homologous positions. When all the landmarks are placed, we can call the group of landmarks that describe an object as a landmark configuration.
- The landmark configurations are aligned and scaled using a best-fit procedure that minimizes differences between them. This is called Procrustes superimposition. After Procrustes superimpositions, we lose all information about size, and we deal with shape only.
- The Cartesian coordinates (*x*, *y*, *z*) of the aligned/scaled objects are called Procrustes coordinates. The Procrustes coordinates of an object define its position in a system, called shape-space.
- The shape-space extends along many dimensions, since each object has many landmarks. For 3-dimensional objects, the number of dimensions of the shape-space is equal to three times the number of landmarks minus seven (degrees of freedom).
- Each object can be considered as a single point in shape-space. The distance between two objects in shape-space is equal to their shape difference.
- The average shape of all the objects is the shape at the center of the shape-space and can be easily computed by averaging the Procrustes coordinates of each landmark.
- To determine the shape patterns of the population, the shape-space is rotated in such a way that its main axes are aligned to the directions of major variability of the population. This is achieved by applying the PCA. PCA describes differences between shapes through determination of the main sources of variability when comparing different shapes.

## Landmark identification and placement

When applying GMM to objects of orthodontic interest, such as bone and soft tissue surfaces, palates, or teeth, we cannot study curves and surfaces directly but we need to place landmarks on them. Thus, the most challenging problems are how many landmarks to place, which criteria (anatomical? geometrical?) to use to place landmarks, how densely should we cover each surface, and finally how can we ascertain that landmarks are *homologous* from one object to the other.

The last question is particularly troublesome because in orthodontics we come across some very extensive areas, such as the cheeks on the facial soft-tissue surface that do not possess any distinguishing/non-ambiguous markers. A similar problem occurs in 2D data, e.g., the outline of the mandible on a lateral cephalogram is a smooth line and there is no anatomical structure to guide us in placing points such as the gonion.

To manage this problem, Bookstein identified three types of landmarks: [18]

▪ Type I landmarks are those that are located at the juxtaposition of anatomical features, such as the confluence of three sutures meeting at a single point. Type I landmarks are defined by features in their immediate vicinity and can be confidently assumed homologous, at least in the anatomical sense.

▪ Type II landmarks are defined as the maxima of curvature of an anatomical structure, e.g., the anterior nasal spine defined as the point of highest curvature of the maxillary outline.

▪ Type III landmarks are defined as points along a curve or surface, in relation to some other more distant structure. For example, the menton is located on the mandibular outline but needs other structures (e.g., the Frankfurt horizontal) or an external vertical direction (e.g., the true vertical) to define its precise location.

Curves or surfaces that do not provide explicit information for precise location of landmarks are ubiquitous. The simple solution is to place landmarks at predetermined intervals along the curve, e.g., at equidistant intervals. Points placed with such a criteria that stay on the curve/surface are called semi-landmarks [19, 20]. The geometry is easier for curves in 2D or 3D but it is not so easy to define semi-landmarks for non-planar surfaces in 3D. In any case, semi-landmarks after first placement are not guaranteed to be 100% homologous.

GMM approach the semi-landmark placement problem by changing their position until the additional shape variability is reduced to the minimum possible. This is achieved by sliding the semi-landmarks in the direction that reduces shape variance but always constraining them on the curve or surface. Once slid (the procedure needs to be repeated at least three times), the semi-landmarks can be considered as homologous points, and the shapes are ready to be analyzed through Principal Component Analysis.

## Anatomical variability

Anatomical variability between subjects in the sample may lead to a debatable controversy: what to do if an anatomical trait (e.g., a cusp, a ridge on a tooth, a ruga on the palate) is absent in one of the individuals? Two solutions are possible: the first one is to ignore it in all subjects. The second is to place the landmark at the same anatomical spot, effectively making the trait zero size. No ideal solution exists. However, we can experience the same problem with conventional measurements, e.g., how do you measure overjet if there are no incisors [13]. In this latter case, the solution would likely be to exclude the patient from the sample, while in the case of geometric morphometrics, it may be acceptable to give to the anatomical trait a “zero” value and include it in calculations, if the missing anatomical trait is not the main clinical question to solve.

## Advantages and disadvantages of GMM

When using GMM, we renounce to have any information on size, as all the shapes are “averaged” and size information is left out of the Procrustes space. This can be seen as a disadvantage as only change in shape patterns can be outlined through GMM. Anyway, this limitation can turn into an advantage. In fact, there is no need to arbitrarily select a special part of the shape to be measured as all parts can be compared as far as a landmark fits the area. While looking at palates, we can get much more information through a GMM procedure rather than with standardized measures.

Another important aspect is that in orthodontics we normally compare anatomical features between patients and controls, assuming that controls are more regular or “normal.” However, what can be considered normal or not normal, is controversial and of difficult interpretation. With GMM, variation of shapes just comes out from the population, considering all the aspects of the shape, without the need of pre-selecting some parts of the population. Variability analysis through PCA allows to determine shape patterns and can thereafter dictate which measures to take and not vice versa.

When pre-selecting patients with different anatomical features (like in the example of palates collected from oral breathers and standard breathers), GMM has the role to underline the source of differences between the two samples. If the samples are really different as for their space entities, they should appear clusterized, as at least the group with pathologic problem (oral breathers) represents an extreme of the population.

## Findings

### GMM and palatal surfaces

Two samples of mouth-breathing (19 subjects, 12 females and 7 males, with a mean age of 8.5 ± 1.6 years) and nose-breathing subjects (16 subjects, 8 females and 8 males, mean age 8.5 ± 1.7 years) in the mixed dentition have been already selected and studied for anatomical differences in terms of palatal vault linear dimensions, surface, and volume [21]. Having 3D data such as surface and volumes does not give a much better insight on palatal shape changes, as the value of the surface or of the volume is a mere number that does not tell where is the source of variability between patients. GMM was thus applied to describe the shape of the palates. The use of GMM allows to understand the changes of shape not only in preselected areas (i.e., molars and canine transverse distance, palatal height, palatal depth) but virtually in any point of the surface where homologous landmarks and semi-landmarks were positioned, thus allowing for a more comprehensive understanding of changes in shape.

**Fig. 1:**
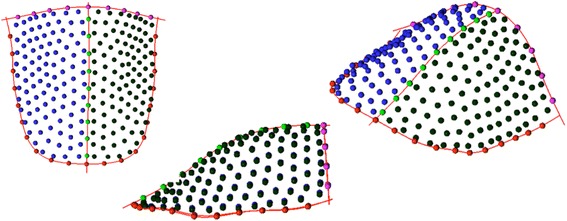
The template of 240 landmarks and semi-landmarks used to study the palatal shape

Palatal vaults were digitized through a template representing a dataset of homologous landmarks. The template was created with Viewbox (Viewbox 4, dHAL software, Kifissia, Greece) and applied for all the patients [9] (Fig. 1).

Three boundary lines were defined for each palate: a midline passing on the median raphe, a U-shaped line passing apical to the gingival sulci of each tooth, and a posterior boundary, perpendicular to the plane defined by the previous line at the height of the mesial contact points of upper first molars. The extremes of the lines were digitized and provisional semi-landmarks were used to approximate the curvature of each line along the surfaces. Semi-landmarks were then automatically placed and distributed at equal distances along the lines and on the palatal surface delimited by the described lines. A total of 240 landmarks and semi-landmarks were registered for each palate. The average of all the dataset was then calculated and used as a reference to allow the semi-landmarks of each palate to become more and more homologous through a repeated (×3) sliding procedure that minimizes the differences between each palate and the average template.

**Fig. 2:**
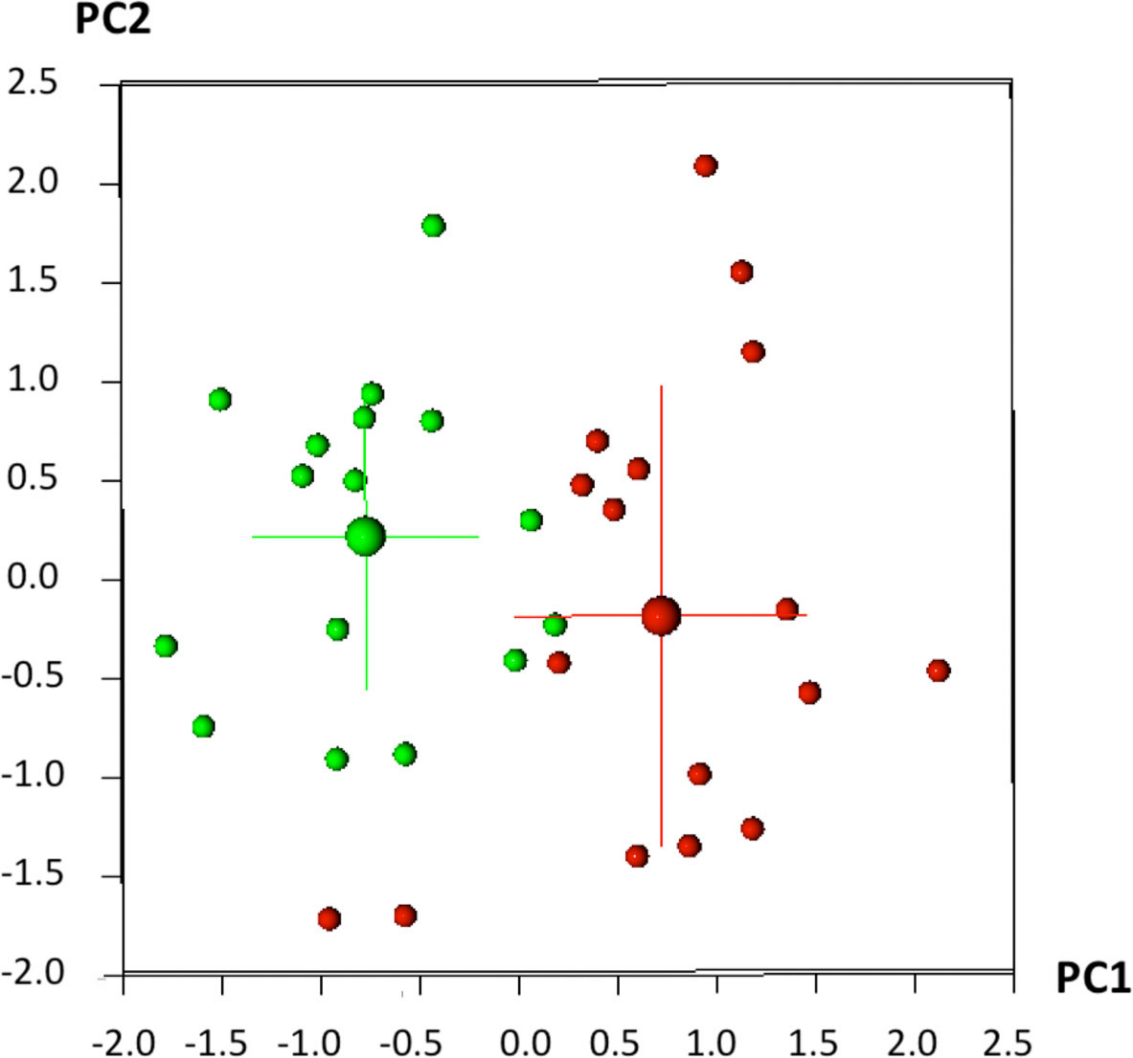
Clusterized results: in green, nose breather/controls; in red, mouth breathers/patients. On the *x*-axis PC1, on the *y*-axis PC2 from Principal Component Analysis calculation

**Fig. 3:**
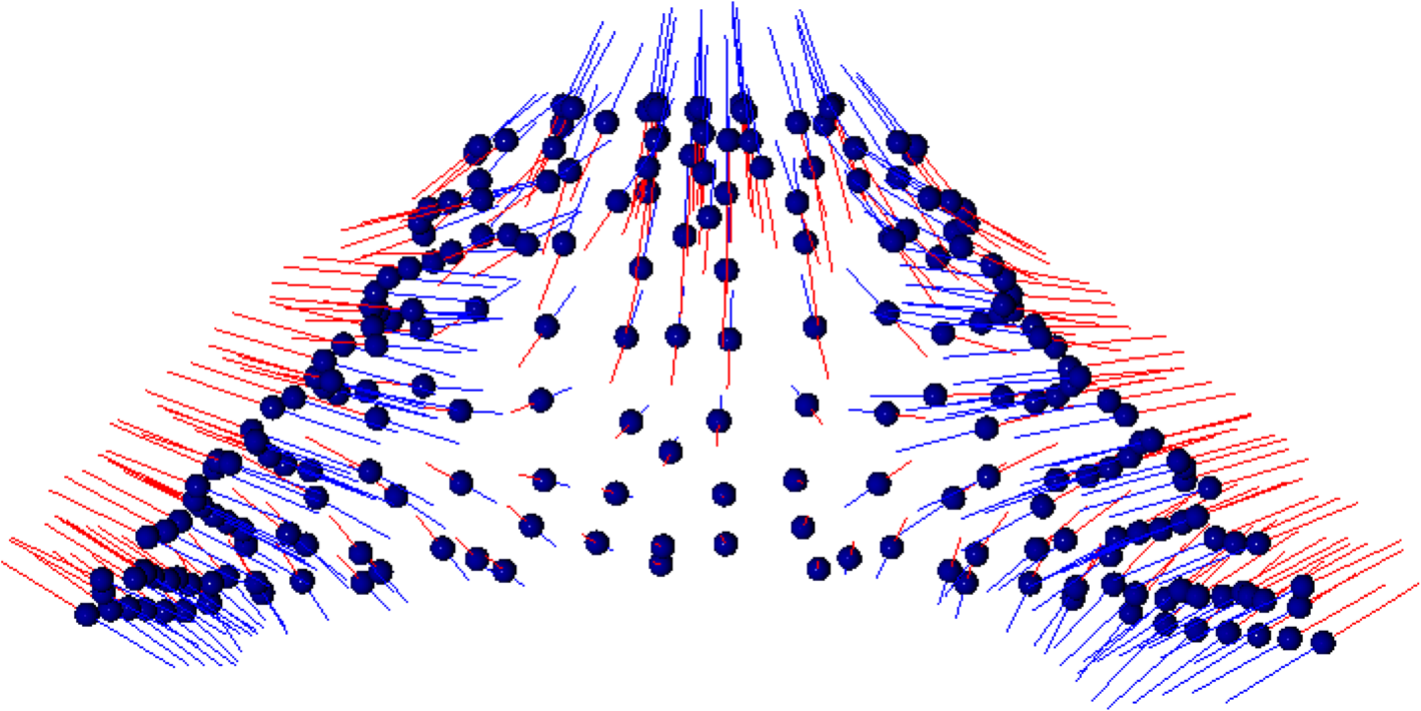
On the left, PC1 resulted into 50% of variability source: the main difference consisted of a larger and shorter palatal vault height vs a narrower and higher palate. Blue lines show the trend of mouth breathers; red lines show the trend of nasal breathers

Principal Component Analysis (PCA) was calculated for all the palates of both samples. PCA allows to determine the source of variability of the shape in between a sample. A Procrustes distance between groups means a test was applied and a *p* = 0.10 was calculated. Even though not statistically significant, PCA results showed that the two samples were clusterized (Fig. 2), thus being different in the shape/form space. Fifty-five percent (PC1) of the morphological differences may be explained through a difference in transverse dimension in the lateral side of the palate (narrower for oral breather and larger for normal breathers Fig. 3) and in the height of the palatal vault, while 15% (PC2) of the differences may be explained with a difference only in the vertical dimension.

**Fig. 4:**
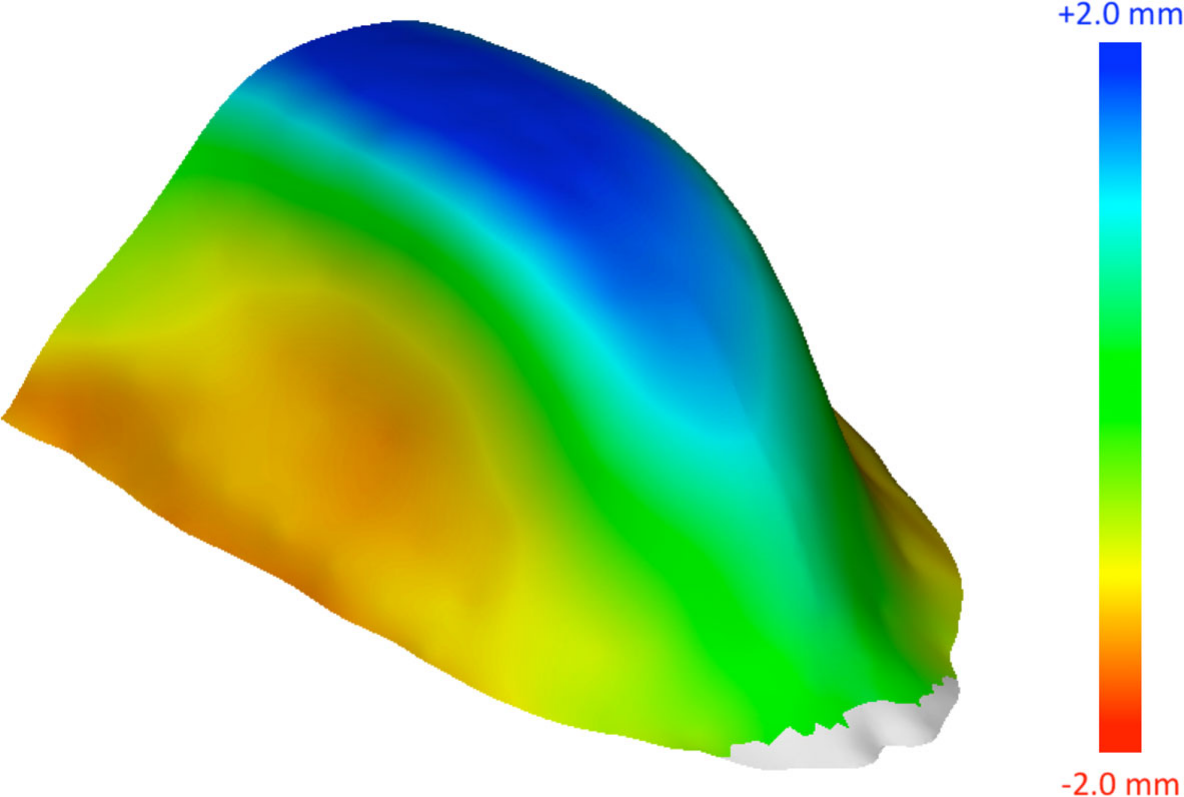
Colorimetric maps showing the differences in shape after superimposition between the average palate of mouth breathers and nasal breathers: the palate is narrower and higher in mouth breathers

Finally, the average morphology of the palate was calculated for each group (the average shape does not give information on shape size after performing Procrustes superimposition). A mesh was adapted through warping to the average palates both for mouth and nose breather. Warping consists of “sticking” a 3D surface to a point configuration (in this case the average configuration for both groups). A colorimetric map was used to visually present differences between the two shapes and make them easily identifiable from a clinical point of view (Fig. 4). The morphological analysis is coherent with the linear, surface, and volumetric results reported in the previous paper [14]. Moreover, it is consistent with the clinical observation of narrow and higher palatal vault in oral-breathers when compared to the palatal vault of nasal-breathers. GMM can be a useful tool to visually illustrate and describe with a scientific method the differences in palatal shape between subjects/samples.

## Conclusions

Geometric morphometrics (GMM) can be used as a useful tool to describe the three-dimensional shape of surfaces of orthodontic interest. A general introduction to the theoretical principles of how to apply GMM is provided.

Variability can be measured through the Principal Component Analysis (PCA) and can lead to the identification of shape patterns and sources of variability of the shape, independently from changes in size.
